# N6-methyladenosine modification of KLF2 may contribute to endothelial-to-mesenchymal transition in pulmonary hypertension

**DOI:** 10.1186/s11658-024-00590-w

**Published:** 2024-05-13

**Authors:** Kang Kang, Jingjing Xiang, Xingshi Zhang, Yuting Xie, Mengting Zhou, Le Zeng, Junhao Zhuang, Jiahao Kuang, Yuanyuan Lin, Bozhe Hu, Qianmin Xiong, Qing Yin, Qiang Su, Xiaoyun Liao, Jun Wang, Yanqin Niu, Cuilian Liu, Jinglin Tian, Deming Gou

**Affiliations:** 1 Department of Biochemistry and Molecular Biology, Shenzhen University Medical School, Shenzhen, 518060 Guangdong People’s Republic of China; 2 Shenzhen Key Laboratory of Microbial Genetic Engineering, Vascular Disease Research Center, College of Life Sciences and Oceanography, Guangdong Provincial Key Laboratory of Regional Immunity and Disease, Carson International Cancer Center, School of Medicine, Shenzhen University, Shenzhen, 518060 China

**Keywords:** Pulmonary arterial hypertension, Transcription factor, Methylation, Epigenetics, Epithelial-mesenchymal transition

## Abstract

**Background:**

Pulmonary hypertension (PH) is a progressive disease characterized by pulmonary vascular remodeling. Increasing evidence indicates that endothelial-to-mesenchymal transition (EndMT) in pulmonary artery endothelial cells (PAECs) is a pivotal trigger initiating this remodeling. However, the regulatory mechanisms underlying EndMT in PH are still not fully understood.

**Methods:**

Cytokine-induced hPAECs were assessed using RNA methylation quantification, qRT-PCR, and western blotting to determine the involvement of N6-methyladenosine (m^6^A) methylation in EndMT. Lentivirus-mediated silencing, overexpression, tube formation, and wound healing assays were utilized to investigate the function of METTL3 in EndMT. Endothelial-specific gene knockout, hemodynamic measurement, and immunostaining were performed to explore the roles of METTL3 in pulmonary vascular remodeling and PH. RNA-seq, RNA Immunoprecipitation-based qPCR, mRNA stability assay, m^6^A mutation, and dual-luciferase assays were employed to elucidate the mechanisms of RNA methylation in EndMT.

**Results:**

The global levels of m^6^A and METTL3 expression were found to decrease in TNF-α- and TGF-β1-induced EndMT in human PAECs (hPAECs). METTL3 inhibition led to reduced endothelial markers (CD31 and VE-cadherin) and increased mesenchymal markers (SM22 and N-cadherin) as well as EndMT-related transcription factors (Snail, Zeb1, Zeb2, and Slug). The endothelial-specific knockout of *Mettl3* promoted EndMT and exacerbated pulmonary vascular remodeling and hypoxia-induced PH (HPH) in mice. Mechanistically, METTL3-mediated m^6^A modification of kruppel-like factor 2 (KLF2) plays a crucial role in the EndMT process. KLF2 overexpression increased CD31 and VE-cadherin levels while decreasing SM22, N-cadherin, and EndMT-related transcription factors, thereby mitigating EndMT in PH. Mutations in the m^6^A site of KLF2 mRNA compromise KLF2 expression, subsequently diminishing its protective effect against EndMT. Furthermore, KLF2 modulates SM22 expression through direct binding to its promoter.

**Conclusions:**

Our findings unveil a novel METTL3/KLF2 pathway critical for protecting hPAECs against EndMT, highlighting a promising avenue for therapeutic investigation in PH.

**Supplementary Information:**

The online version contains supplementary material available at 10.1186/s11658-024-00590-w.

## Introduction

Pulmonary hypertension (PH) is a severe pulmonary vascular disorder characterized by pulmonary vascular remodeling, which can lead to pulmonary artery stenosis and eventual right ventricle (RV) failure [1].

Emerging evidence indicates that endothelial-to-mesenchymal transition (EndMT), a process involving the transition of endothelial cells (ECs) from a cobblestone to a spindle-like phenotype, is critical in EC dysfunction and subsequent vascular remodeling [2, 3]. During EndMT, several zinc finger transcription factors, such as Snail, Slug, Zeb1, and Zeb2, are activated, serving as repressors or activators to orchestrate the decline of endothelial markers, such as CD31, VE-cadherin, and vWF, and the rise of mesenchymal markers, such as N-cadherin, SM22, and α-SMA [4]. Frid et al. initially identified the involvement of TGF-β1 in inducing EndMT in ECs isolated from bovine pulmonary arteries [5]. Moreover, hypoxia has been shown to promote EndMT via upregulation of myocardin [6]. In monocrotaline pyrrole (MCTP)-induced experimental PH models, pulmonary vascular endothelial cells have been observed to transform toward EndMT, leading to neointimal formation [7]. Good et al. further highlighted the significance of EndMT in PH by demonstrating an increased coexpression of vWF and α-SMA in the lung vessels of hypoxia/sugen-treated mice [8]. A multitude of mechanisms, including hypoxia [6], inflammation [9], aberrant BMPR2 signaling [10], and oxidative stress [11], have been implicated in the induction of EndMT. In addition, epigenetic modifications, such as DNA methylation of the eNOS promoter [12], P300-dependent histone acetylation [13], and the involvement of the long non-coding RNA ANRIL [14] and NORAD [15], are associated with EC dysfunction. Despite these advancements in understanding epigenetic regulation of EC behavior, knowledge regarding the role of RNA methylation in PH-associated EndMT remains nascent.

N6-methyladenosine (m^6^A) is the most prominent RNA modification extensively investigated in recent studies [16–18]. The modulation of m^6^A methylation predominantly involves three categories of effector proteins: METTL3, METTL14, and WTAP as writers; FTO and ALKBH5 as erasers; and YTHDF1/2/3 and YTHDC1/2 as readers [19–21]. Accumulating evidence suggests that RNA methylation plays a crucial role in PH progression. METTL14-mediated m^6^A methylation leads to mRNA decay of Grb-2-related adaptor protein (GRAP), promoting pulmonary vascular resistance [22]. Likewise, YTHDF1 recognizes the m^6^A mark on Foxm1 mRNA, facilitating pulmonary vascular changes and fibrosis [23]. In contrast, depletion of YTHDF1 attenuates PH development by identifying m^6^A on MAGED1 mRNA [24]. Moreover, RNA modifications are also essential in regulating endothelial dysfunction. Elevation of C-C motif chemokine receptor 10 (CCR10) decreases m^6^A methylation, promoting endothelial injury [25]. Similarly, human cytomegalovirus accelerates endothelial apoptosis through METTL3 and YTHDF3-mediated m^6^A modification [26]. Kong et al. recently identified that m^6^A methylation on TRPC6 enhances hypoxia-mediated EndMT in rat PAECs through activating calcineurin/NFAT signaling [27]. Despite these insights, the specific role of m^6^A in EndMT during PH is not fully understood.

In this study, we elucidate that the global methylation status and the m^6^A RNA methyltransferase METTL3 are downregulated during EndMT in human pulmonary artery endothelial cells (hPAECs) exposed to TNF-α and TGF-β1. METTL3 deficiency triggers EndMT in vitro and exacerbates pulmonary vascular remodeling and PH progression in vivo. Mechanistically, Kruppel-like factor 2 (KLF2), a downstream effector of METTL3, undergoes significant suppression in an m^6^A-dependent manner. Ectopic expression of KLF2 protects hPAECs against EndMT, offering a novel perspective for therapeutic research in PH.

## Materials and methods

### Animal models

Conditional C57BL/6 *Mettl3* knockout mice were created by flanking exons 2/3 in *Mettl3* genomic DNA with *loxP* sites. These mice, termed *Mettl3*^*flox/flox*^ (*Mettl3*^*fl/fl*^), were then crossed with a tamoxifen-inducible *Cdh5* promoter-driven Cre line (*Cdh5-Cre*^*ERT2*^) to generate endothelial-specific *Mettl3* knockout mice (*Cdh5-Cre*^*ERT2*^*;Mettl3*^*fl/fl*^). Littermates bearing the *Mettl3*^*fl/fl*^ genotype served as controls. Tamoxifen was delivered via intraperitoneal injection (20 mg/kg/day × 5 days, i.p.) a week prior to exposure to hypoxic conditions. Male mice, aged 8 weeks, from both the *Cdh5-Cre*^*ERT2*^*;Mettl3*^*fl/fl*^ and *Mettl3*^*fl/fl*^ genotypes, were randomly assigned into two groups and housed in either normoxic (21% O_2_) or hypoxic (10% O_2_) environments for 3 weeks. After anesthetizing with 10% chloral hydrate (0.3–0.4 mL/100 g), these mice underwent hemodynamics and histological analysis.

### Cell culture

Human pulmonary artery endothelial cells (hPAECs) were purchased from ScienCell Research Laboratories (ScienCell, 3100, San Diego, USA) and Human embryonic kidney (HEK293T) cells were sourced from the American Type Culture Collection (ATCC, CRL-11268, Manassas, VA, USA). HEK293T cells were propagated in DMEM (Dulbecco's Modified Eagle Medium, Corning, 10-013-CVR) supplemented with 10% FBS and 1% penicillin–streptomycin (Solarbio, P1400) and maintained in an incubator containing 5% CO_2_. hPAECs were cultured in full ECM (endothelial cell medium, Sciencell, 1001) supplemented with 5% FBS, 1% endothelial cell growth addition (ECGS), and 1% penicillin–streptomycin, and were also incubated under the aforementioned conditions. For hypoxia experiments, hPAECs were placed in a special hypoxia incubator infused with a gas mixture of 5% CO_2_ and nitrogen to obtain 1% oxygen concentration. Oxygen concentration was monitored continuously (Forma 3130; Thermo Scientific, Rockford, IL).

### Mouse endothelial cell isolation

Mouse endothelial cells were isolated as described [28, 29]. In brief, the peripheral lung tissues (about 1.0 mm on the edge of the lung tissue) encompassing microvessels were sheared and minced. The tissues were individually digested at 37 °C for 30 min in phosphate buffer saline (PBS) supplemented with collagenase (2 mg/mL), papain (0.4 mg/mL), bovine serum albumin (BSA, 2 mg/mL) and DNase I (15 mg/mL). The homogenate was filtered through a 40 μm cell strainer. The cell suspension was collected and incubated with mouse CD31 microbeads (Miltenyi Biotec, 130-097-418) at 4 °C for 15 min. The microbeads were washed with PBS supplemented with 2 mM EDTA and 0.5% BSA using a MS column (Miltenyi Biotec, 130-042-201) and a magnetic separator (Miltenyi Biotec, 130-042-303), and then used for total RNA isolation.

### Cytokine treatment

Overnight cultured hPAECs at approximately 30% confluence were treated with 5 ng/mL TNF-α and 5 ng/mL TGF-β1 in the ECM and incubated for 3 days. The cell lines were utilized between the 4th and 6th passages.

### Plasmid construction and lentivirus production

Lentiviral shRNA and overexpression vectors were constructed based on a modified Lenti-X vector (Clontech), with the U6 and cytomegalovirus (CMV) promoter directing the expression of shRNA and cDNA, respectively. The coding sequences (CDSs) of METTL3 (NM_019852.5) and KLF2 (NM_016270.4) were cloned into the lentivirus vector to generate the pLV-OE-METTL3 and pLV-OE-KLF2 expression vectors, respectively. Additionally, both the wild-type (WT) and m^6^A mutant KLF2 3′-UTR were inserted into the pLV-OE-KLF2 vector downstream of the KLF2 CDS. The lentivirus particles were prepared in HEK293T cells through transfection of three distinct plasmids at a ratio of 2:1:3, namely, (i) psPAX2 (Addgene), (ii) pCMV-VSV-G (Addgene), and (iii) the lentiviral vector. After transfection for 72 h, the virus in the culture medium was harvested, filtered through 0.45-μm polyvinylidene difluoride filters (Millipore, SLH033) and preserved at − 80 °C. For lentiviral infection, 1 × 10^5^ cells at 40–50% confluence were infected with 2–3 × 10^5 transduction units (TU) of lentiviruses in the presence of polybrene at a final concentration of 5 μg/mL. The primers used are listed in Additional file 1: Table S1.

### Quantitative real‑time polymerase chain reaction (qRT‑PCR)

Total RNA was extracted utilizing RNAiso Plus (TaKaRa, Dalian, China). The SYBR Green method was employed for cDNA synthesis with oligo (dT) and random primers as previously described [30]. The relative mRNA expression levels normalized to β-actin were calculated using the 2^−ΔΔCt^ method. The primers used are listed in Additional file 1: Table S2.

### Western blotting

Both cells and tissue were lysed using cold RIPA buffer (50 mmol/L Tris·HCl, pH 7.5, 150 mmol/L NaCl, 1% NP-40, 0.25% sodium deoxycholate, and 1 mmol/L EDTA) supplemented with protease inhibitor cocktail (Roche, Mannheim, Germany). Protein concentration was determined with the Bicinchoninic Acid Protein Assay Kit (Thermo Fisher Scientific). Equal amounts of protein (~ 30 μg) were subjected to SDS-PAGE and transferred to PVDF membranes. Following blocking with 5% BSA in Tris-buffered Saline-Tween 20 (TBST; 20 mmol/L Tris·HCl, pH 7.6, 150 mmol/L NaCl, and 0.1% Tween 20), the membranes were incubated with primary antibodies overnight at 4 °C and then with horseradish peroxidase-conjugated goat anti-rabbit IgG or goat anti-mouse IgG secondary antibodies at room temperature for 1 h. The protein bands were visualized using a chemiluminescence detection module (Pierce Biotechnology, Rockford, IL) and captured on a Chemiluminescence Intelligent Image Workstation (BLT GelView 6000Plus, China). The antibodies used are listed in Additional file 1: Table S4.

### Tube formation assay

hPAECs infected with either shNC or shMETTL3 lentivirus were seeded on Matrigel (BD, New Jersey, USA) in 24-well plates at a density of 1 × 10^5^ cells/well. After a 6-h incubation at 37 °C, tube morphological features were assessed and quantified using ImageJ software.

### Wound healing assay

To evaluate cell migration, a wound healing assay was conducted. Confluent hPAECs infected with either shNC or shMETTL3 lentiviruses were gently scratched within 24-well plates. The cells were then cultivated in 0.2% FBS-ECM, and images of the wounded areas were captured at 0, 12, 24, and 36 h. The wound closure percentage was calculated using the formula: migration area (%) = (original area – remaining area)/original area × 100. ImageJ was employed for image analysis.

### Luciferase reporter assays

The JASPAR database (https://jaspar.genereg.net/) was used to analyze the promoter sequences. SM22 expression regulation was assessed using a dual reporter gene assay comprising a firefly luciferase construct and a Renilla luciferase reference construct pRL-TK (Promega, Madison, WI). A 1.2 kb SM22 promoter segment was PCR-amplified from human genomic DNA and inserted into the pGl4.3 vector between *Xho*I and *Mlu*I. HEK293T cells stably expressing either shNC or shKLF2 were cotransfected with 150 ng of pGl4.3-SM22-WT (or pGl4.3-SM22-Mut) and 5 ng of pRL-TK reporter using PEI reagent. After 48 h of transfection, dual luciferase activities were measured with a Dual-luciferase Reporter System (E1980, Promega, Madison WI USA) using a Lumat LB9508 luminometer (Berthold, Bad Wildbad, Germany), with firefly luciferase activity normalized to the Renilla luciferase activity for each sample.

### Hemodynamic measurements

The transonic catheter was utilized to determine the mean right ventricular systolic blood pressure (RVSP). These readings were documented using the MP150 system and subsequently analyzed by the AcqKnowledge 4.2 software package (BIOPAC Systems, Inc.). Following the hemodynamic evaluations, the animals were euthanized. For assessment of right ventricular remodeling, the heart was dissected, and the right ventricular hypertrophy index (RVHI) was determined by calculating the weight ratio of the right ventricle (RV) to the sum of the left ventricle plus ventricular septum (LV + S).

### Morphological and histological analysis

For morphological evaluations, lung sections from paraffin-embedded samples were stained with hematoxylin–eosin (HE). To quantify the medial wall thickness, 10–15 pulmonary arteries with diameters of 50–100 μm were inspected from each mouse. The percentage of wall thickness and wall area was calculated using the following formulas: relative wall thickness = (outer perimeter − inside perimeter)/outer perimeter, and relative wall area = (outer area − inside area)/outer area. In the immunostaining process, the lung sections were rehydrated in alcoholic baths after dewaxing. After antigen retrieval using citric acid buffer (pH 6.0), primary antibodies against α-SMA (1:200, GB111364, Servicebio), METTL3 (1:200, GB114688, Servicebio), and either Cy3-labeled (1:1000, Jackson ImmunoResearch Labs) or Alexa Fluor 488-labeled secondary antibodies (1:1000, Abcam) were applied. Each section was then counterstained with DAPI Fluoromount-G.

### RNA sequencing and data analysis

cDNA libraries were prepared using the VAHTS Stranded mRNA-seq Library Prep Kit for Illumina (NR612, Vazyme, Inc., Nanjing, China) and subsequently sequenced on the Illumina NovaSeq 6000 platform. After quality control and read mapping to the human reference genome (RGCh38/hg38), differential expression analysis between control and treated samples was performed using the DESeq2 R package (1.20.0). A padj-value < 0.05 and fold change (FC) ≥ 2 were the criteria for significantly differential expression. Differentially expressed genes were further analyzed for pathway involvement using the Kyoto Encyclopedia of Genes and Genomes (KEGG). A KEGG term with Q values ≤ 0.05 was considered to be significantly enriched.

### MeRIP-qPCR

For methylated RNA Immunoprecipitation-based qPCR (MeRIP-qPCR), total RNAs from hPAECs infected with either shNC or shMETTL3 lentiviruses were utilized. Briefly, 10 μg of total RNA was combined with 1 μg of anti-m^6^A antibody (Cat. no. 202 003 Synaptic Systems) or the respective control IgG (ab172730, Abcam) in 200 μL 1 × IP buffer. This mixture was incubated at 4 °C for 2 h, followed by a 2-h incubation with protein A/G magnetic beads (Sera-Mag, USA) at 4 °C. The immunoprecipitated RNAs were then eluted by treatment with Thermolabile Proteinase K (#P8111S, NEB) in reverse transcription buffer at 37 °C for 30 min, and then at 55 °C for 10 min to inactivate the enzyme. The eluted RNAs were directly subjected to RT and qPCR analysis. Additionally, 0.5 μg of the initial total RNA was reserved as input. The relative enrichment of m^6^A in each sample was calculated by normalization to this input control.

### RNA methylation quantification

The methylation quantification of the purified RNA was assayed using the EpiQuik m^6^A RNA Methylation Quantification Kit (P-9005, EpiGentek) according to the manufacturer’s instructions.

### mRNA stability assay

Cells were treated with actinomycin D (5 μg/mL) for the indicated time and the mRNA levels at each time point were analyzed by qRT-PCR.

### Statistical methods

The data were analyzed using GraphPad Prism version 8.3.0 (GraphPad Software, Inc., San Diego, CA). All data are presented as the mean value ± standard deviation (mean ± SD). For comparisons between two groups, a two-tailed unpaired t test was employed. Differences among three or more groups were analyzed using one-way ANOVA followed by Tukey's multiple comparisons test. A *P* value of < 0.05 was considered statistically significant.

## Results

### METTL3 was downregulated in EndMT of hPAECs

**Fig. 1:**
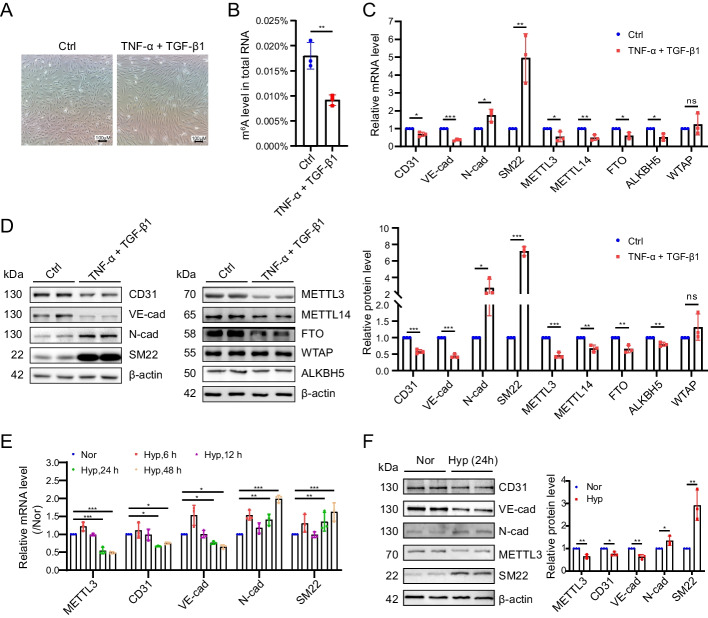
METTL3 was diminished during EndMT. **A** Morphological change in EndMT in hPAECs. hPAECs were treated with cytokines (TNF-α and TGF-β1) for 3 days, with PBS serving as the control (Ctrl). **B** The m^6^A levels in total RNA were analyzed in the Ctrl and cytokine-treated hPAECs (*n* = 3). **C**, **D** The mRNA (**C**) and protein levels of CD31, VE-cadherin (VE-cad), N-cadherin (N-cad), SM22 (left panel, **D**), METTL3, METTL14, FTO, ALKBH5, and WTAP (middle panel, **D**) were detected by qRT-PCR and western blotting. The bar chart depicts the relative protein level (right panel, **D**, *n* = 3). **E**, **F** hPAECs were cultured in normoxia (Nor) for 24 h as control or hypoxia (Hyp) for 6, 12, 24, and 48 h, and the mRNA (**E**) and protein (**F**) levels of METTL3, CD31, VE-cad, N-cad and SM22 were detected by qRT-PCR and western blotting. The bar chart depicts the relative protein level (*n* = 3). β-Actin was used as an internal reference for qRT-PCR and as a loading control for western blotting. A two-tailed unpaired t test (**B**–**D**, **F**) or one-way ANOVA followed by Tukey's multiple comparisons test (**E**) was used to estimate the significance. Statistical significance is denoted by *P < 0.05, **P < 0.01, and ***P < 0.001

To elucidate the impact of m^6^A modification in endothelial-to-mesenchymal transition (EndMT), we analyzed the global m^6^A methylation status and the expression of RNA methylation-associated enzymes, METTL3, METTL14, WTAP, FTO, and ALKBH5, in cytokine (TNF-α and TGF-β1)-induced EndMT of hPAECs (Fig. 1A). Our findings revealed that the m^6^A level of total RNA in the cytokine-treated hPAECs was significantly reduced compared to that in the untreated control (Ctrl) group (Fig. 1B). Concurrently, a significant reduction in the endothelial markers CD31 and VE-cadherin, coupled with a pronounced increase in the mesenchymal markers SM22 and N-cadherin, was observed (Fig. 1C, D), suggesting a robust EndMT in the cytokine-treated hPAECs. Among the enzymes examined, METTL3 exhibited the most significant reduction during EndMT. While METTL14, FTO, and ALKBH5 also showed decreased expression, albeit to a lesser degree, WTAP levels remained stable (Fig. 1C, D). Further investigation into hypoxia-induced EndMT in hPAECs also revealed a marked reduction in METTL3 expression as hypoxia duration increased (Fig. 1E, F). These findings suggest that METTL3 may play a critical role in mediating EndMT in PH, leading us to focus on METTL3 in our subsequent research.

### METTL3 inhibition promotes EndMT and leads to endothelial dysfunction in vitro

**Fig. 2:**
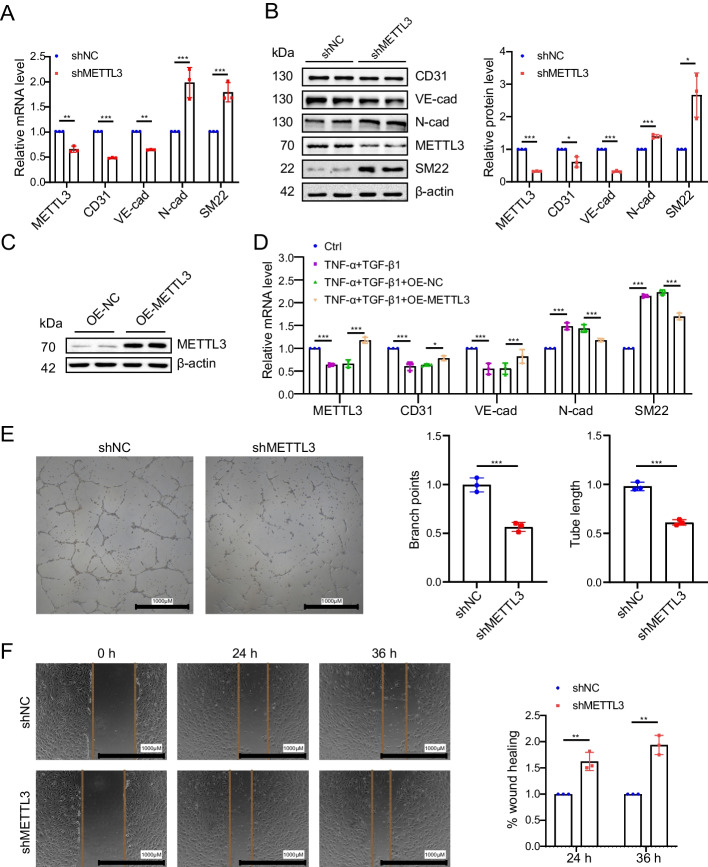
METTL3 knockdown triggers EndMT and endothelial dysfunction in vitro. **A**, **B** The mRNA (**A**) and protein levels (**B**) of METTL3, CD31, VE-cadherin (VE-cad), N-cadherin (N-cad), and SM22 were assessed by qRT-PCR and western blotting in hPAECs infected with shMETTL3 lentivirus compared to the control group shNC (*n* = 3). Bar chart shows the relative protein levels (right panel, **B**). **C** The protein levels of METTL3 were assessed by western blotting in hPAECs overexpressing METTL3 coding sequence (OE-METTL3) compared to the control group (OE-NC) overexpressing a green fluorescent protein (*n* = 3). **D** The mRNA levels of METTL3, CD31, VE-cad, N-cad, and SM22 were detected by qRT-PCR in the Ctrl and cytokine-treated hPAECs infected with lentivirus of OE-METTL3 or OE-NC (*n* = 3). β-Actin was used as an internal reference for qRT-PCR and as a loading control for western blotting. **E** Representative images of angiogenesis tube formation assays (left). The number of branch points (middle) and tube length (right) were calculated using ImageJ (*n* = 3). Scale bar represents 1000 μm. **F** Representative images of the wound healing assay are shown (left) (*n* = 3). The percent of wound closure (right) was analyzed using ImageJ and calculated using the following formula: migration area (%) = (original area – remaining area)/the original area × 100. Scale bar represents 1000 μm. Bar chart elucidates the changes in wound width at 24 and 36 h (h). A two-tailed unpaired t test (**A**, **B**, **E**, **F**) or one-way ANOVA followed by Tukey's multiple comparisons test (**D**) was used to estimate the significance. Statistical significance is denoted by *P < 0.05, **P < 0.01, and ***P < 0.001

To investigate the biological role of METTL3 downregulation in EndMT in hPAECs, we employed lentivirus-mediated shRNA to target METTL3. METTL3 expression was significantly reduced in hPAECs infected with shMETTL3 lentivirus (Fig. 2A, B). This suppression of METTL3 reduced the endothelial markers CD31 and VE-cadherin but elevated the mesenchymal markers N-cadherin and SM22 (Fig. 2A, B). A rescue experiment demonstrated that overexpression of METTL3 reversed cytokine (TNF-α and TGF-β1)-induced decrease of METTL3 (Fig. 2C, D). Additionally, METTL3 overexpression mitigated the cytokine-induced reduction in CD31 and VE-cadherin levels, while concurrently attenuating the elevation of SM22 and N-cadherin (Fig. 2C, D). A tube formation assay suggested that METTL3 elimination impaired the angiogenic potential of hPAECs relative to the control (Fig. 2E). Furthermore, a wound healing assay indicated enhanced cell migration with METTL3 inhibition compared to the control (Fig. 2F). Collectively, these results underline that METTL3 plays an important role in preventing EndMT and endothelial dysfunction in hPAECs.

### Endothelial-specific Mettl3 ablation aggravates hypoxia-induced PH in vivo

**Fig. 3:**
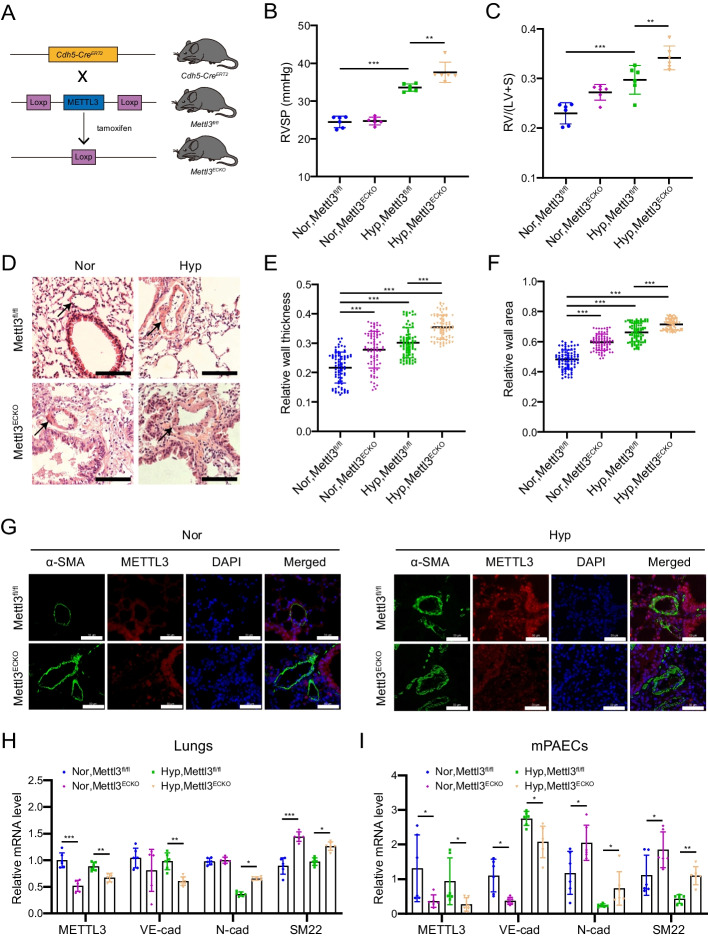
Endothelial-specific knockout of *Mettl3* augments EndMT and PH in vivo. **A** Depiction of the conditional endothelial-specific *Mettl3* loss-of-function mouse model. **B**, **C** Measurement of right ventricular systolic pressure (RVSP) in mmHg by right heart catheterization (**B**) and evaluation of right ventricular hypertrophy by the weight ratio of the right ventricle to the sum of the left ventricle plus ventricular septum (RV/(LV + S)) (**C**) in both *Cdh5-Cre*^*ERT2*^*;Mettl3*^*fl/fl*^* (Mettl3*^*ECKO*^*)* knockout mice and *Mettl3*^*fl/fl*^ control mice (*n* = 6). **D** Representative images of hematoxylin and eosin (HE)-stained lung sections from both *Mettl3*^*ECKO*^ and *Mettl3*^*fl/fl*^ mice. Scale bars, 100 μm. **E**, **F** Pulmonary arterial wall thickness analysis for 6 mice (*n* = 80 per group). The relative wall thickness (**E**) was determined as (outer perimeter − inside perimeter)/outer perimeter (left), while the relative wall area (**F**) was deduced from (outer area − inside area)/outer area (right). **G** Representative dual immunostaining using METTL3 and α-SMA antibodies for pulmonary arteries in both mouse types (*n* = 6). Scale bars, 50 μm. **H**, **I** The mRNA levels of METTL3, VE-cadherin (VE-cad), N-cadherin (N-cad), and SM22 in mouse lungs (**H**) and PAECs (mPAECs) (**I**) were evaluated by qRT-PCR (*n* = 6). β-Actin was used as an internal reference for qRT-PCR. Nor: normoxia; Hyp: hypoxia. A one-way ANOVA followed by Tukey's multiple comparisons test was used to estimate the significance. Statistical significance is denoted by *P < 0.05, **P < 0.01, and ***P < 0.001

To elucidate the role of METTL3 in the progression of pulmonary hypertension, we generated endothelial-specific *Mettl3* knockout mice (Fig. 3A). Subsequently, *Cdh5-Cre*^*ERT2*^*;Mettl3*^*fl/fl*^ (*Mettl3*^*ECKO*^) knockout mice and *Mettl3*^*fl/fl*^ control mice were exposed to either hypoxia (10% O_2_) or normoxia (21% O_2_) for 3 weeks. Hemodynamic assessments revealed a significant elevation in right ventricular systolic pressure (RVSP) and right ventricular hypertrophy index (RVHI) in *Mettl3*^*fl/fl*^ mice under hypoxia compared to normoxia (Fig. 3B, C). Compared to hypoxic *Mettl3*^*fl/fl*^ mice, *Mettl3*^*ECKO*^ mice displayed higher increases in both RVSP and RVHI under hypoxic conditions (Fig. 3B, C), suggesting that *Mettl3* knockout in endothelial cells aggravated hypoxia-induced PH hemodynamic alterations. Histological analysis also exhibited pronounced pulmonary arterial wall thickening and remodeling in *Mettl3*^*ECKO*^ mice compared to *Mettl3*^*fl/fl*^ mice (Fig. 3D–F). Immunostaining using an antibody against α-SMA further validated this thickening in *Mettl3*^*ECKO*^ mice (Fig. 3G).

In addition, qRT-PCR assays demonstrated a significant reduction of METTL3 expression in both the lungs and PAECs of *Mettl3*^*ECKO*^ mice compared to *Mettl3*^*fl/fl*^ mice (Fig. 3H, I). Trace levels of METTL3 were detectable in knockout mouse PAECs, likely because the METTL3 gene continued to generate truncated transcripts following the deletion of several exons, specifically exons 2, 3, and also exon 4, potentially as a result of alternative splicing. Sequence analysis revealed that frameshift mutations within these truncated transcripts resulted in premature termination codons, effectively preventing the production of functional METTL3 protein (Additional file 1: Fig. S1; Table S3). Endothelial-specific knockout of *Mettl3* in mice induced a marked downregulation of VE-cadherin alongside an upregulation of N-cadherin and SM22 in both the lungs and PAECs of *Mettl3*^*ECKO*^ mice compared to *Mettl3*^*fl/fl*^ mice (Fig. 3H, I). Taken together, these findings indicate that endothelial-specific deletion of *Mettl3* boosts EndMT and exaggerates pulmonary vascular remodeling and PH.

### Inhibition of METTL3 disrupts inflammatory signaling by targeting KLF2 in an m^6^A-dependent manner

**Fig. 4:**
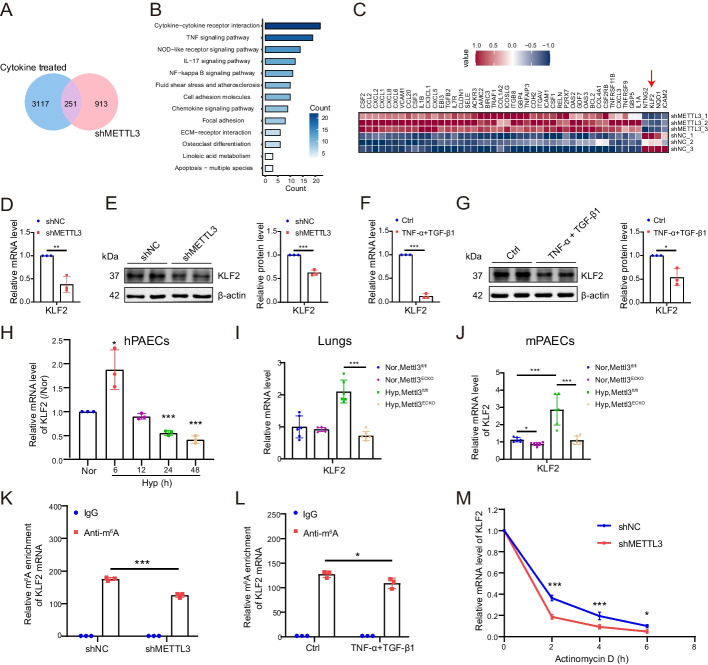
METTL3 inhibition induced inflammatory signaling dysregulation and resulted in KLF2 downregulation in an m^6^A-dependent manner. **A** Intersection of DEGs from METTL3-silenced hPAECs with those induced by cytokines (TNF-α and TGF-β1) is visualized in a Venn diagram. **B** KEGG pathway analysis highlighting the predominant pathways among intersecting DEGs. **C** A heatmap exhibiting the most significant DEGs in METTL3-silenced hPAECs from transcriptome sequencing (*n* = 3). **D**, **E** The mRNA and protein levels of KLF2 in shNC and shMETTL3 hPAECs were determined by qRT-PCR (**D**) and western blotting (**E**) (*n* = 3). **F**, **G** The mRNA and protein levels of KLF2 in hPAECs treated with cytokines or PBS control (Ctrl) were determined by qRT-PCR (**F**) and western blotting (**G**) (*n* = 3). **H** the mRNA levels of KLF2 were detected by qRT-PCR in hPAECs cultured in normoxia (Nor) for 24 h as control or hypoxia (Hyp) for 6, 12, 24, and 48 h (*n* = 3). **I**, **J** The mRNA expression levels of KLF2 in mouse lungs (**I**) and PAECs (mPAECs) (**J**) were evaluated by qRT-PCR (*n* = 6). **K**, **L** MeRIP-qPCR analysis indicates the m^6^A levels on KLF2 after METTL3 inhibition (**K**) or cytokine treatment (**L**). The fold change in m^6^A enrichment is defined as (IP/input) % of shMETTL3 divided by (IP/input) % of shNC. **M** shNC and shMETTL3 hPAECs were treated with actinomycin D (5 μg/mL) for the indicated times. The expression of KLF2 was examined by qRT-PCR. β-Actin was used as an internal reference for qRT-PCR and as a loading control for western blotting. A two-tailed unpaired t test (**D**–**G**, **K**–**M**) or one-way ANOVA followed by Tukey's multiple comparisons test (**H**–**J**) was used to estimate the significance. Statistical significance is denoted by *P < 0.05, **P < 0.01, and ***P < 0.001

To elucidate the mechanisms by which METTL3 modulates EndMT, transcriptomic analyses were performed on hPAECs transfected with shMETTL3 or control shRNA (shNC), and on those treated with cytokines (TNF-α and TGF-β1) versus PBS (as a control, Ctrl). Quantitative analysis identified 913 differentially expressed genes (DEGs) in METTL3-silenced hPAECs [padj-value < 0.05; fold change (FC) ≥ 2], consisting of 433 up- and 480 down-regulated genes (Additional file 1: Fig. S2A). In cytokine-treated hPAECs, 3117 DEGs were observed [padj-value < 0.05; fold change (FC) ≥ 2], comprising 1838 up- and 1279 down-regulated genes (Additional file 1: Fig. S2B). An intersection of these DEG datasets identified 251 overlapping genes (Fig. 4A). KEGG analysis of these genes highlighted several inflammation-related pathways, including cytokine-cytokine receptor interaction, TNF signaling pathway, NF-κB signaling pathway, fluid shear stress and atherosclerosis, and chemokine signaling (Fig. 4B). The most significant DEGs in these pathways are detailed in Fig. 4C.

Among the identified DEGs, KLF2 is known to exhibit anti-inflammatory and antithrombotic activities in endothelial cells [31, 32]. qRT-PCR and western blotting analyses confirmed the dramatic downregulation of KLF2 in hPAECs following METTL3 knockdown (Fig. 4D, E). TNF-α/TGF-β1-treated hPAECs also displayed markedly decreased KLF2 mRNA and protein levels (Fig. 4F, G). In addition, KLF2 downregulation was observed during the progression of hypoxia-induced EndMT in hPAECs (Fig. 4H). Intriguingly, the qRT-PCR assay highlighted a significant decrease in KLF2 in the lungs and PAECs of *Mettl3*^*ECKO*^ mice compared to *Mettl3*^*fl/fl*^ mice under hypoxic conditions (Fig. 4I, J). These findings suggest that elimination of *Mettl3* promotes EndMT and facilitates PH through modulating KLF2.

Subsequently, we investigated the mechanism by which METTL3 and cytokines regulate the expression of KLF2. MeRIP-qPCR analysis revealed that KLF2 mRNA was immunoprecipitated from hPAECs using an anti-m^6^A antibody but not with the IgG control (Fig. 4K). METTL3 silencing resulted in decreased m^6^A modification on KLF2, indicating the important role of METTL3 in regulating KLF2 expression through m^6^A methylation. Furthermore, the m^6^A enrichment of KLF2 was also reduced during cytokine-induced EndMT in hPAECs (Fig. 4L). Additionally, to explore the mechanism through which METTL3-specific m^6^A methylation controls KLF2 expression, Control and METTL3-deficient hPAECs were subjected to treatment with the transcriptional inhibitor actinomycin D. This treatment demonstrated that suppression of METTL3 leads to a decrease in KLF2 mRNA stability, indicating that METTL3-specific m^6^A methylation controls KLF2 expression by regulating KLF2 mRNA stability (Fig. 4M). This finding is consistent with Mo’s report that a higher m^6^A level promotes KLF2 mRNA stability [33].

Collectively, our findings suggest that METTL3 potentially modulates hPAEC EndMT through KLF2 in an m^6^A-dependent manner.

### The functional role of KLF2 in modulating EndMT

**Fig. 5:**
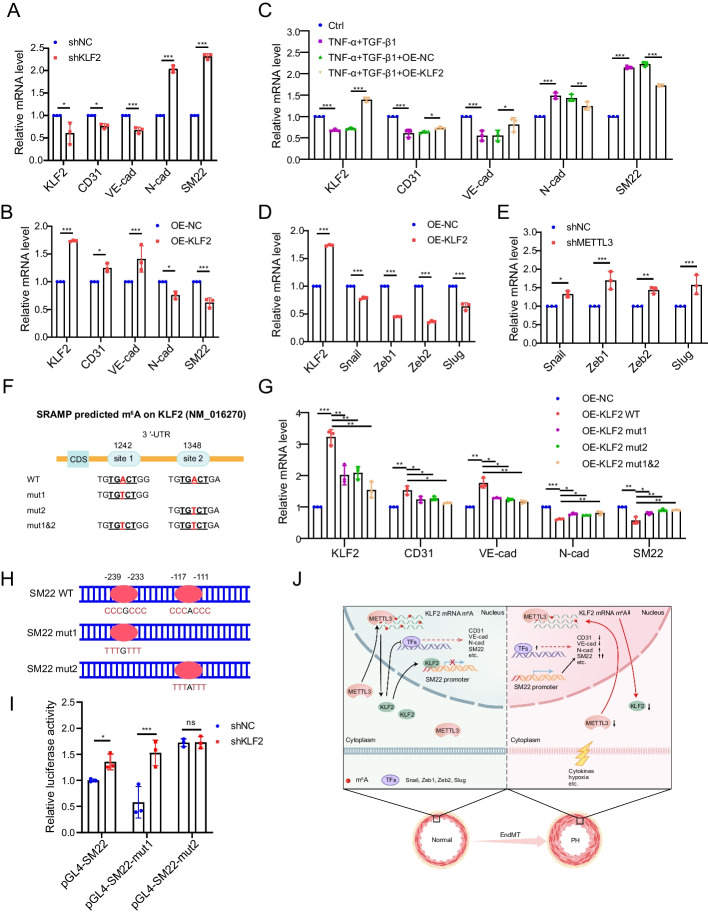
Upregulation of KLF2 protects hPAECs against EndMT. **A**, **B** The mRNA expression levels of CD31, VE-cadherin (VE-cad), N-cadherin (N-cad), and SM22 in hPAECs were assessed by qRT-PCR following KLF2 silencing (**A**) and KLF2 overexpression (**B**) (*n* = 3). **C** The mRNA levels of KLF2, CD31, VE-cad, N-cad and SM22 were detected by qRT-PCR in the Ctrl and cytokine-treated hPAECs infected with lentivirus overexpressing the KLF2 coding sequence (OE-KLF2) or OE-NC (*n* = 3). **D**, **E** The mRNA expression levels of the transcription factors Snail, Zeb1, Zeb2, and Slug in hPAECs were assessed by qRT-PCR under KLF2 overexpression (**D**) and METTL3 inhibition (**E**) (*n* = 3). **F**, **G** hPAECs were infected with lentiviruses overexpressing KLF2 CDS-3′-UTR with either wild type (WT) or mutant (mut, A-to-T mutation) m^6^A sites (**F**), and the mRNA levels of KLF2, CD31, VE-cad, N-cad, and SM22 were detected by qRT-PCR (**G**). β-Actin was used as an internal reference for qRT-PCR. **H** Potential binding sites of KLF2 on the SM22 promoter were identified using the JASPAR database (http://jaspar.genereg.net/). **I** HEK293T cells expressing either shNC or shKLF2 were transfected with luciferase reporter plasmids containing the wild-type SM22 promoter or its variants with specified mutations (site 1, − 233 to − 239; site 2, − 111 to − 117). Luciferase activity was quantified 48 h after transfection (*n* = 3). A two-tailed unpaired t test (**A**, **B**, **D**, **E**, **I**) or one-way ANOVA followed by Tukey's multiple comparisons test (**C**, **G**) was used to estimate the significance. Statistical significance is denoted by *P < 0.05, **P < 0.01, and ***P < 0.001. **J** A schematic of m^6^A modification of KLF2 regulating EndMT. Under normal physiological conditions, METTL3-driven m^6^A modification maintains KLF2 expression, sustaining the levels of CD31 and VE-cadherin while inhibiting the transcription factors Snail, Zeb1, Zeb2, and Slug, as well as the mesenchymal markers N-cadherin and SM22, in PAECs. However, when exposed to pathological stimuli, such as pro-inflammatory cytokines or hypoxia, the decrease in METTL3 downregulates m^6^A methylation, leading to diminished KLF2. This results in the upregulation of EndMT-associated transcription factors and mesenchymal markers, thereby promoting EndMT and the onset of PH

To elucidate the functional role of KLF2 in modulating EndMT, we silenced KLF2 in hPAECs using lentivirus-mediated shRNA. Knockdown of KLF2 resulted in decreased expression of CD31 and VE-cadherin (Fig. 5A, B). In contrast, KLF2 overexpression enhanced their expression in hPAECs. Moreover, silencing KLF2 elevated the levels of SM22 and N-cadherin, whereas overexpressing KLF2 inhibited them in hPAECs (Fig. 5A, B). A rescue assay revealed that KLF2 overexpression counteracted the cytokine (TNF-α and TGF-β1)-induced decrease in CD31 and VE-cadherin levels, simultaneously reducing the increase in SM22 and N-cadherin (Fig. 5C). To reveal the underlying mechanism by which KLF2 modulates EndMT, we assessed the expression levels of the EndMT-related transcription factors Snail, Zeb1, Zeb2, and Slug, which play critical roles in EndMT progression [34, 35]. Our findings indicate that these transcription factors were significantly decreased in hPAECs upon KLF2 overexpression (Fig. 5D). Consistently, METTL3 silencing led to an increase in their levels (Fig. 5E).

We utilized an m^6^A site predictor SRAMP (http://www.cuilab.cn/sramp/) and identified two high-scoring m^6^A modification sites (sites 1242 and 1348) within the 3′-UTR of KLF2 mRNA (Additional file 1: Fig. S3). To investigate the effects of m^6^A modifications on KLF2 in the context of EndMT, we incorporated these potential m^6^A sites into lentiviruses overexpressing KLF2 by replacing adenosine with thymine at each site (Fig. 5F). Analysis of hPAECs infected with these lentiviruses revealed that KLF2 expression levels in cells with either single or double mutant KLF2 3′-UTR were lower than those in cells with the wild-type (WT) KLF2 3′-UTR (Fig. 5G). Additionally, mutations at either or both m^6^A sites led to decreased CD31 and VE-cadherin levels and increased SM22 and N-cadherin levels, compared to the WT KLF2 3′-UTR. These results indicate that METTL3 regulates KLF2 expression and its impact on EndMT through an m^6^A-dependent mechanism.

Using the JASPAR database (https://jaspar.genereg.net/), we identified two potential KLF2 binding sites within the promoter region of SM22 (site 1, − 233 to − 239; site 2, − 111 to − 117; Fig. 5H). The dual-luciferase reporter assay revealed that silencing KLF2 increased luciferase activity in comparison to the shNC group, indicating the suppressive effect of KLF2 on SM22 promoter activity (Fig. 5I). Interestingly, mutating binding site 2, but not site 1, led to an increase in luciferase activity, indicating the prevention of KLF2 binding to the SM22 promoter regardless of the levels of KLF2 (Fig. 5I). These results suggest that KLF2 might protect cells against EndMT through both indirect regulation of EndMT-relevant transcription factors and direct modulation of SM22 transcriptional activity.

## Discussion

Pulmonary hypertension (PH) is a progressive disorder characterized by endothelial dysfunction, abnormal proliferation of smooth muscle cells, and infiltration of inflammatory cells, resulting in vascular remodeling [36]. EndMT is pivotal in the pathogenesis of endothelial dysfunction in PH.

Several molecular mechanisms have been implicated in the onset of EndMT including hypoxia [6], aberrant inflammatory and BMPR2 regulation [9, 10], reactive oxygen species [11], and various forms of epigenetic regulation such as DNA methylation [37], histone modification [38], and non-coding RNA [14, 39]. In this study, we observed a significant downregulation of METTL3 during EndMT in hPAECs. Our experiments demonstrated that silencing METTL3 significantly induces EndMT in vitro. Further, through the use of endothelial-specific knockout mouse models, we confirmed that genetic deletion of *Mettl3* leads to exacerbated endothelial cell dysfunction, pulmonary vascular remodeling, and the progression of PH in vivo. Mechanistically, METTL3 modulates EndMT via regulating KLF2 in an m^6^A-dependent manner. Consequently, our findings highlight the crucial role of RNA epigenetics in the pathogenesis of PH-associated EndMT.

Kruppel-like factor 2 (KLF2) is a shear stress-sensitive transcription factor that is predominantly expressed in ECs and is essential for maintaining endothelial homeostasis [40, 41]. Inhibition of KLF2 has been shown to induce EndMT in HUVECs [42], whereas enhanced KLF2 expression can counteract brain-derived neurotrophic factor-induced EndMT in HUVECs by impeding HK1-mediated glucose metabolism reprogramming [43]. A missense mutation in KLF2 has been identified in heritable pulmonary arterial hypertension (HPAH) [44], highlighting its significant role in PH progression. Furthermore, KLF2 levels are decreased in the lung tissues of PH patients [39]. KLF2 overexpression leads to an increase in exosomal miR-181a-5p and miR-324-5p, which in turn diminishes ECs apoptosis, NFκB signaling, VEGF-driven proliferation, and pulmonary vascular remodeling through targeting the Notch4 and ETS1 pathways [39]. In our research, we found that knockdown of KLF2 induces EndMT, while its overexpression prevents EndMT in hPAECs. We also revealed that high levels of KLF2 suppress the expression of EndMT-related transcription factors. Collectively, these findings underline the protective role of KLF2 against EC dysfunction.

Various factors have been correlated with the downregulation of KLF2 expression in PH, including interrupted BMPR2 activity [44], pro-inflammatory cytokines [45], and impaired AMP-activated kinase activity [46]. Our research reveals that METTL3-mediated m^6^A modification plays a key role in the regulation of KLF2 expression. Silencing METTL3 leads to decreases in both m^6^A modification and KLF2 mRNA levels. Furthermore, mutations in the m^6^A site of KLF2 mRNA compromise KLF2 expression, thereby diminishing its protective effect against EndMT. Analogously, Mo et al. reported that atorvastatin-induced reduction in FTO promoted KLF2 and eNOS expression via m^6^A modification, affecting endothelial function in atherosclerosis [33]. These findings collectively emphasize the key role of m^6^A RNA methylation in regulating EndMT through modulating KLF2 expression.

Research has demonstrated that KLF proteins exert strict regulatory control over the expression of mesenchymal markers. Specifically, the loss of KLF4 elevates the expression of collagens, VCAM-1, SMA, and SM22 in human lung ECs [47]. Moreover, KLF4 represses SM22 transcription during the phenotypic transition of smooth muscle cells via cooperatively binding with pELK-1 to the SM22 promoter [48]. In this study, we revealed that KLF2 inhibition enhanced the levels of the mesenchymal markers SM22 and N-cadherin, while KLF2 overexpression led to their reduction. Furthermore, KLF2 directly bound to the SM22 promoter, suppressing its transcription. These results underscore the critical role of KLFs in regulating the expression of mesenchymal markers, particularly SM22, in various contexts.

Contrary to our observations, Kong et al. recently reported an upregulation of METTL3 in hypoxia-induced EndMT, which facilitates EndMT via the activation of the TRPC6/calcineurin/NFAT signaling pathways [27]. The discrepancy may be explained by differences in experimental models (hPAECs versus rat PAECs), the specific downstream targets of METTL3 (KLF2 versus TRPC6), and cellular responses to stimuli, including TNF-α/TGF-β1 and hypoxia. Notably, our study detected an increase in METTL3 expression in hPAECs in early hypoxia (at 12 h), which significantly declined with prolonged exposure (at 24 h and 48 h) (Fig. 1E). Additionally, our transcriptome analysis revealed selective upregulation within the TRPC6/calcineurin/NFAT pathway, with only TRPC6/NFATC4 in cytokine-treated hPAECs and NFATC1 in METTL3-silenced hPAECs showing significant upregulation (Additional file 1: Fig. S4). This finding contrasts with the results in Chunchu Kong’s study.

Additionally, Qin et al. observed elevated METTL3 expression in hypoxic conditions, which subsequently enhances pulmonary artery smooth muscle cells (PASMCs) proliferation and migration by modulating the PTEN/PI3K/Akt signaling pathway [49]. The contrasting observations suggest that the role of METTL3 varies among different cell types involved in PH, such as PAECs versus PASMCs. Moreover, the stage of PH progression, as well as specific downstream targets and signaling pathways influenced by METTL3 (KLF2 versus PTEN), can further lead to diverse outcomes. This highlights the nuanced nature of epigenetic regulation in complex diseases such as PH. Future research is imperative to delineate the conditions under which targeting the METTL3/KLF2 pathway could offer the most advantageous therapeutic outcomes. This entails investigating diverse models of PH, analyzing patient samples across various stages of the disease, and examining the impact of other epigenetic and environmental factors.

In this study, we also observed dysregulation of METTL14, FTO, and ALKBH5 during cytokine-treated EndMT. While our study highlights the significance of METTL3 in the m^6^A modification of KLF2 and its implications for EndMT in PH, the contributions of METTL14, FTO, and ALKBH5 present an intriguing avenue for future research. Understanding the intricate balance between m^6^A methylation and demethylation on KLF2 by these enzymes could unravel new dimensions in the epigenetic regulation of PH and identify novel therapeutic targets. Additionally, we focused on RVSP and RVHI as primary indicators of PH hemodynamics in mouse models, due to their direct relevance to PH pathophysiology and widespread recognition as markers of PH progression. A more detailed assessment of hemodynamics, encompassing cardiac output and total pulmonary vascular resistance, would enhance understanding of the effects of epigenetic modifications on PH.

## Conclusion

In conclusion, our research unveils a novel METTL3/KLF2 pathway crucial for safeguarding hPAECs against EndMT. Under normal arterial conditions, METTL3-mediated m^6^A methylation of KLF2 mRNA ensures its optimal expression and functionality, which consequently inhibits the detrimental activation of EndMT-relevant transcription factors such as Snail, Zeb1, Zeb2, and Slug, along with mesenchymal markers including N-cadherin and SM22. In contrast, stimuli such as pro-inflammatory cytokines or hypoxia diminish METTL3 levels, resulting in reduced m^6^A methylation and consequently decreased KLF2 expression. This downregulation of KLF2 triggers an elevation in EndMT-relevant transcription factors and mesenchymal markers, and leads to a concomitant decrease in the endothelial markers CD31 and VE-cadherin. Such alterations fuel the progression of EndMT and, ultimately, PH (Fig. 5J). These insights link METTL3-mediated m^6^A modification of KLF2 with EndMT, offering a more comprehensive view of the molecular landscape of PH.

### Supplementary Information

**Additional file 1: Table S1.** Primers used for plasmid construction. **Table S2.** Primers used for qRT-PCR. **Table S3.** Primers used for sequencing. **Table S4.** Antibodies used for western blotting. **Figure S1.** Sequence analysis of *Mettl3* knockout. **Figure S2.** Differentially expressed genes in hPAECs. **Figure S3.** Prediction of KLF2 mRNA m^6^A sites was performed using SRAMP. **Figure S4.** TRPC6/calcineurin/NFAT pathway in cytokine-treated and METTL3-silenced hPAECs.

## Data Availability

NGS data have been deposited in the NCBI Sequence Read Archive (SRA) and are available through SRA accession numbers PRJNA1018467 and PRJNA1026885.

## Abbreviations

CMV: Cytomegalovirus
EC: Endothelial cell
EndMT: Endothelial-to-mesenchymal transition
HPH: Hypoxia-induced PH
KEGG: Kyoto Encyclopedia of Genes and Genomes
KLF: Kruppel-like factor
m^6^A: N6-methyladenosine
MeRIP: Methylated RNA immunoprecipitation
PAEC: Pulmonary artery endothelial cell
PBS: Phosphate-buffered saline
PH: Pulmonary hypertension
RV: Right ventricle
RVHI: Right ventricular hypertrophy index
RVSP: Right ventricular systolic blood pressure
