# Supplementary material for: N6-methyladenosine modification of KLF2 may contribute to endothelial-to-mesenchymal transition in pulmonary hypertension
Source: Cell Mol Biol Lett. 2024 May 13;29:69. doi: 10.1186/s11658-024-00590-w (PMC11089701; doi:10.1186/s11658-024-00590-w)
Supplement: Supplementary file 1 — Additional file 1: Table S1. Primers used for plasmid construction. Table S2. Primers used for qRT-PCR. Table S3. Primers used for sequencing. Table S4. Antibodies used for western blotting. Figure S1. Sequence analysis of Mettl3 knockout. Figure S2. Differentially expressed genes in hPAECs. Figure S3. Prediction of KLF2 mRNA m6A sites was performed using SRAMP. Figure S4. TRPC6/calcineurin/NFAT pathway in cytokine-treated and METTL3-silenced hPAECs. [file 11658_2024_590_MOESM1_ESM.docx]

**N6-methyladenosine modification of KLF2 may contribute to endothelial-to-mesenchymal transition in pulmonary hypertension**

Kang Kang^1#^, Jingjing Xiang^1#^, Xingshi Zhang^1#^, Yuting Xie^1^, Mengting Zhou^1^, Le Zeng^2^, Junhao Zhuang^1^, Jiahao Kuang^1^, Yuanyuan Lin^1^, Bozhe Hu^1^, Qianmin Xiong^1^, Qing Yin^1^, Qiang Su^2^, Xiaoyun Liao^2^, Jun Wang^2^, Yanqin Niu^2^, Cuilian Liu^2^, Jinglin Tian^2^ and Deming Gou^2*^

Data Supplement

1. Table S1
2. Table S2
3. Table S3
4. Table S4
5. Figure S1
6. Figure S2
7. Figure S3
8. Figure S4

**Table S1. Primers used for plasmid construction**

| pGl4.3-hSM22 Pro-luc2 | SM22 promoter F | 5'- CTAGCCTCGAGCTCCTAGGGCCTTGTTTGGCT -3' |
| --- | --- | --- |
|  | SM22 promoter R | 5'- GTGGCACGCGTTCTGGGTTCGGACAGACAGGAT -3' |
| pGl4.3-hSM22 Pro-luc2-Mut1 | SM22-KLF2-BS1M-F | 5'-CCCCGCCGGCAGCGTGCTTTGTTTCGGCCCGCTCCATCTCC-3' |
|  | SM22-KLF2-BS1M-R | 5'-GGAGATGGAGCGGGCCGAAACAAAGCACGCTGCCGGCGGGG-3' |
| pGl4.3-hSM22 Pro-luc2-Mut2 | SM22-KLF2-BS3M-F | 5'-GAGTCACTGGGGGAGCCGGAAATAAAGAGCGGAGCCGGCTTCCTC-3' |
|  | SM22-KLF2-BS3M-R | 5'-GAGGAAGCCGGCTCCGCTCTTTATTTCCGGCTCCCCCAGTGACTC-3' |
| OE-METTL3 | EcoRI hMETTL3-F1 | 5'-ATCGCGTCTCGAATTCATGTCGGACACGTGGAGC-3' |
|  | BamHI hMETTL3-R1 | 5'-CGATCGTCTCGGATCCCTATAAATTCTTAGGTTTAG-3' |
| OE-KLF2 | EcoRI humanKLF2 F | 5'-AGTCGAATTCATGGCGCTGAGTGAACCCAT-3' |
|  | BamHI humanKLF2 R | 5'-CTGAGGATCCCTACATGTGCCGTTTCATGTGCAG-3' |
| shKLF2 | shKLF2-human-P1 | 5'-ACCGGGCCATTCCAGTGCCATCTGTCTCGAGACA-3' |
|  | shKLF2-human-P2 | 5'-AAAAGGCCATTCCAGTGCCATCTGTCTCGAGACA-3' |
|  | shKLF2-human-P3 | 5'-GATGGCACTGGAATGGCC-3' |
| ShMETTL3 | shMettl3-human-P1 | 5'-ACCGCGTCAGTATCTTGGGCAAGTTCTCGAGAAC-3' |
|  | shMettl3-human-P2 | 5'-AAAACGTCAGTATCTTGGGCAAGTTCTCGAGAAC-3' |
|  | shMettl3-human-P3 | 5'-TTGCCCAAGATACTGACG-3' |
| mouse genotype identification | Mettl3-*loxP*-F1 | 5'-CAAACCTTTACTGTGCTTCCATGA-3' |
|  | Mettl3-*loxP*-R1 | 5'-ATAAACACCAGGCCCTTGGAATAC-3' |
|  | Mettl3-*loxP*-F2 | 5'-CCTCCTTCCCCAGATGAAACTGT-3' |
|  | Mettl3-*loxP*-R2 | 5'-CGTCATCGAAGACCAAACTAACAT-3' |
|  | Cre-F | 5'-AATGCTTCTGTCCGTTTGC-3' |
|  | Cre-R | 5'-ACCAGAGTCATCCTTAGCG-3' |

**Table S2. Primers used for qRT-PCR**

| human CDH5-F | 5'-TTGGAACCAGATGCACATTGAT-3' |
| --- | --- |
| human CDH5-R | 5'-TCTTGCGACTCACGCTTGAC-3' |
| human CD31-F | 5'-CCTTCTGCTCTGTTCAAGCC-3' |
| human CD31-R | 5'-GGGTCAGGTTCTTCCCATTT-3' |
| human CDH2-F | 5'-GTGCATGAAGGACAGCCTCT-3' |
| human CDH2-R | 5'-CCACCTTAAAATCTGCAGGC-3' |
| human SM22-F | 5'-AGTGCAGTCCAAAATCGAGAAG-3' |
| human SM22-R | 5'-CTTGCTCAGAATCACGCCAT-3' |
| hrmMETTL3-F | 5'-CCAGGAGCTTGCTCTTACACA-3' |
| hrmMETTL3-R | 5'-GGTCAGCCATCACAACTGCA-3' |
| hrmMETTL14-F | 5'-CATGCTAATGTTGACATTGAC-3' |
| hrmMETTL14-R | 5'-CGTTGGTCCAACTGTGAGCCA-3' |
| hrmALKBH5-F | 5'-CAGGAAGACAAGATTAGATGCA-3' |
| hrmALKBH5-R | 5'-GCAGCATCAGGGTCTGCCTT-3' |
| hrmFTO-F | 5'-GAAGCTAAGAAACTGAGGCT-3' |
| hrmFTO-R | 5'-TTCAGCTGCCACTGCTGATAG-3' |
| hrmWtap-F | 5'-ATCCAGTACCTCAAGCAAGT-3' |
| hrmWtap-R | 5'-CGTAAACTTCCAGGCACTCA-3' |
| human β-actin-F | 5'-AGAGATGGCCACGGCTGCTT-3' |
| human β-actin-R | 5'-ATTTGCGGTGGACGATGGAG-3' |
| human KLF2-F | 5'-CTACACCAAGAGTTCGCATCTG-3' |
| human KLF2-R | 5'-CCGTGTGCTTTCGGTAGTG-3' |
| human SLUG-F | 5'-CGAACTGGACACACATACAGTG-3' |
| human SLUG-R | 5'-CTGAGGATCTCTGGTTGTGGT-3' |
| human ZEB1-F | 5'-GATGATGAATGCGAGTCAGATGC-3' |
| human ZEB1-R | 5'-ACAGCAGTGTCTTGTTGTTGT-3' |
| human ZEB2-F | 5'-CAAGAGGCGCAAACAAGCC-3' |
| human ZEB2-R | 5'-GGTTGGCAATACCGTCATCC-3' |
| human SNAIL-F | 5'-ACAAGCACCAAGAGTCCG-3' |
| human SNAIL-R | 5'-ATGGCAGTGAGAAGGATGTG-3' |
| mouse-Klf2- F | 5'-TGGAGCTGCTGGAGGCCAAGCC-3' |
| mouse-Klf2- R | 5'-CTTTCGGTAGTGGCGGGTAAGC-3' |
| mr β-actin F | 5'-GTGACGTTGACATCCGTAAAGA-3' |
| mr β-actin R | 5'-GCCGGACTCATCGTACTCC-3' |
| MmCDH2-F | 5'-CGCCATCATCGCTATCCT-3' |
| MmCDH2-R | 5'-CTTCTCCTCCACCTTCTTCAT-3' |
| mouse CD31-F | 5'-CAGCCATTACGGTTATGATG-3' |
| mouse CD31-R | 5'-GCTCAAGGGAGGACACTTCC-3' |
| MmCDH5-F | 5'-CAGAGTCCATCGCAGAGT-3' |
| MmCDH5-R | 5'-AGCCAGCATCTTGAACCT-3' |
| mouse SM22-F | 5'-ACTACCGTGGAGATCCCAA-3' |
| mouse SM22-R | 5'-TTTCTAACTGATGATCTGCC-3' |

**Table S3. Primers used for sequencing**

| mMETTL3-ORF-F1 | 5'-ATGTCGGACACGTGGAGCTCTA-3' |
| --- | --- |
| mMETTL3-ORF-R1 | 5'-CGCTTAGCTTTGTAAGGAAGTG-3' |

**Table S4. Antibodies used for** **western blotting**

| Name | Brand, Catalog number | Dilution |
| --- | --- | --- |
| METTL3 | Abcam，ab195352 | 1:1000 |
| METTL14 | Proteintech，26158-1-AP | 1:1500 |
| FTO | Abcam，ab92821 | 1:1000 |
| ALKBH5 | Proteintech，16837-1-AP | 1:1000 |
| WTAP | Proteintech，10200-1-AP | 1:1000 |
| CDH2 | CST，14215s | 1:1000 |
| CDH5 | CST，2500 | 1:2000 |
| SM22 | Abclonal，A6760 | 1:1000 |
| CD31 | Abcam，ab119339 | 1:1000 |
| KLF2 | Thermo，PA5-40591 | 1:1000 |
| β -actin | Servicebio，GB12001 | 1:2500 |

**Figure S1**

**
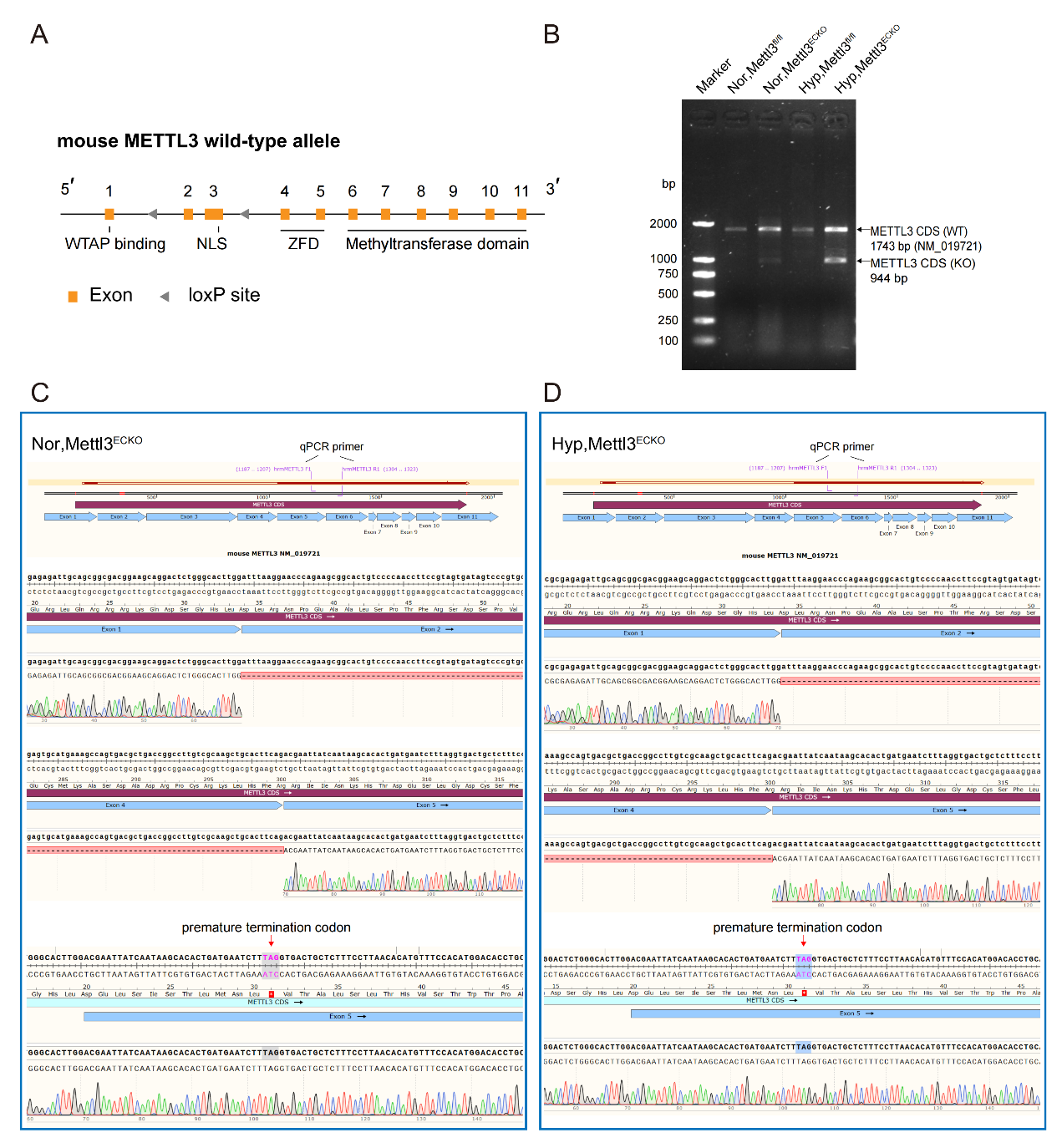
**

**Fig. S1** Sequence analysis of *Mettl3* knockout. **A** *Mettl3* conditional knockout strategy using Cre-*loxP* system. The WTAP-binding domain, nuclear localization signal (NLS), zinc finger domain (ZFD) and methyltransferase domain were shown. **B** The coding sequence (CDS) of *Mettl3* was amplified using RT-PCR from the lung tissues of both *Mettl3^ECKO^* and *Mettl3^fl/fl^* mice, followed by analysis with agarose gel electrophoresis. **C, D** Sequencing analysis of the *Mettl3* CDS was conducted for the mouse of Nor, *Mettl3^ECKO^* (**C**) and Hyp, *Mettl3^ECKO^* (**D**). In addition to exon 2 and 3, exon 4 was also deleted in *Mettl3* CDS, possibly due to alternative splicing. This resulted in the creation of a premature termination codon at exon 5. The sequencing primers used are listed in Additional file 1: Table S3.

**Figure S2**


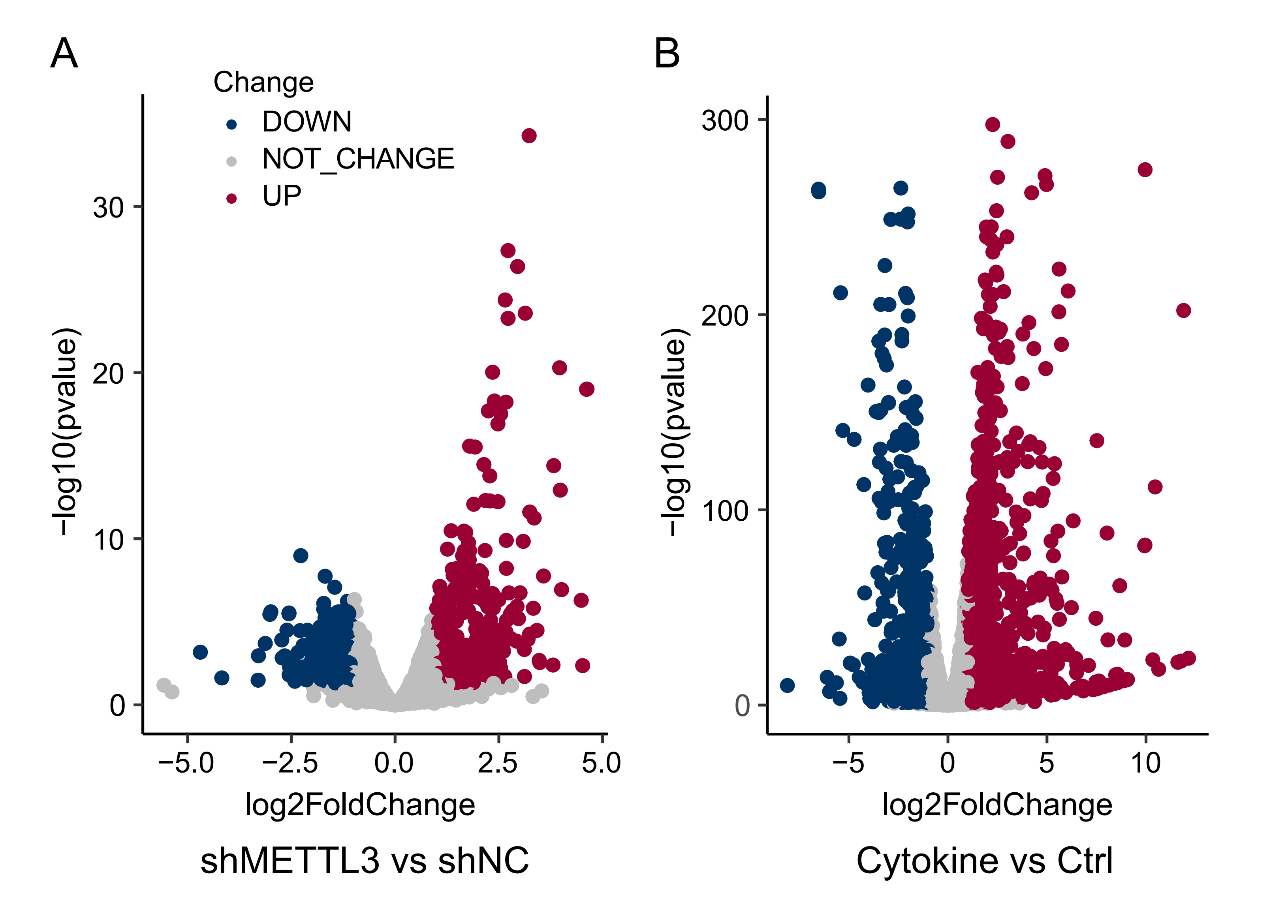


**Fig. S2** Differentially expressed genes in hPAECs. **A** Transcriptome analysis of METTL3-silenced hPAECs. Volcano plot of differentially expressed genes (DEGs) in hPAECs infected with either shMETTL3 or shNC lentiviruses. **B** Transcriptome analysis of cytokine-treated hPAECs. Volcano plot of DEGs in hPAECs treated with cytokines (TNF-α and TGF-β1) or with PBS as the control group (Ctrl). The horizontal axis represents mean log2FoldChange; the vertical axis represents -log10(p-value) of DEseq2; Red color dots stand for upregulation genes. Blue color dots stand for downregulation genes.

**Figure S3**


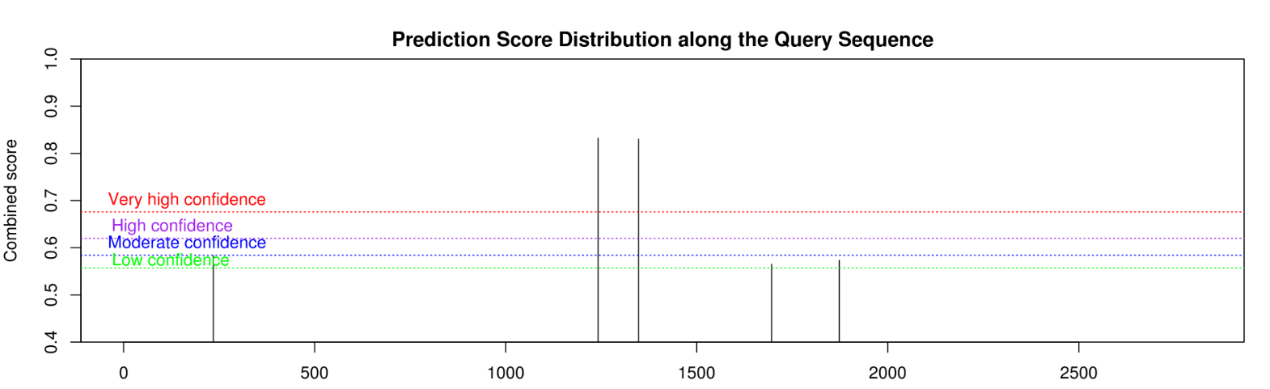


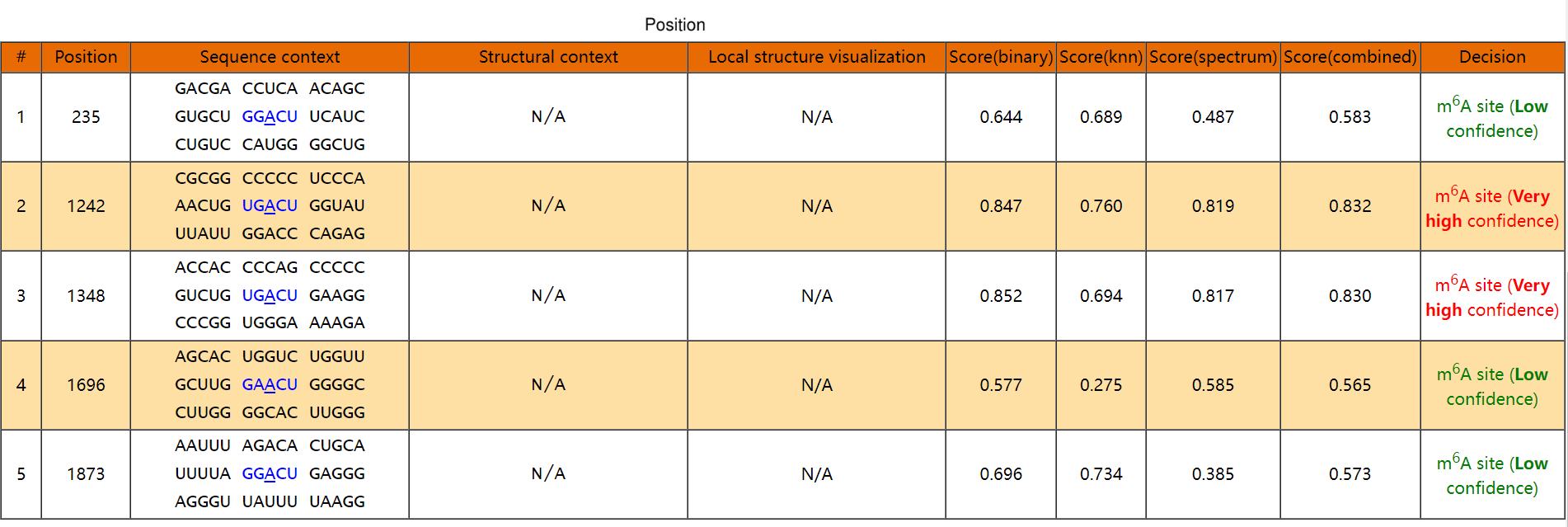


**Fig. S3** Prediction of KLF2 mRNA m^6^A sites was performed using SRAMP (<http://www.cuilab.cn/sramp/>).

**Figure S4**

**
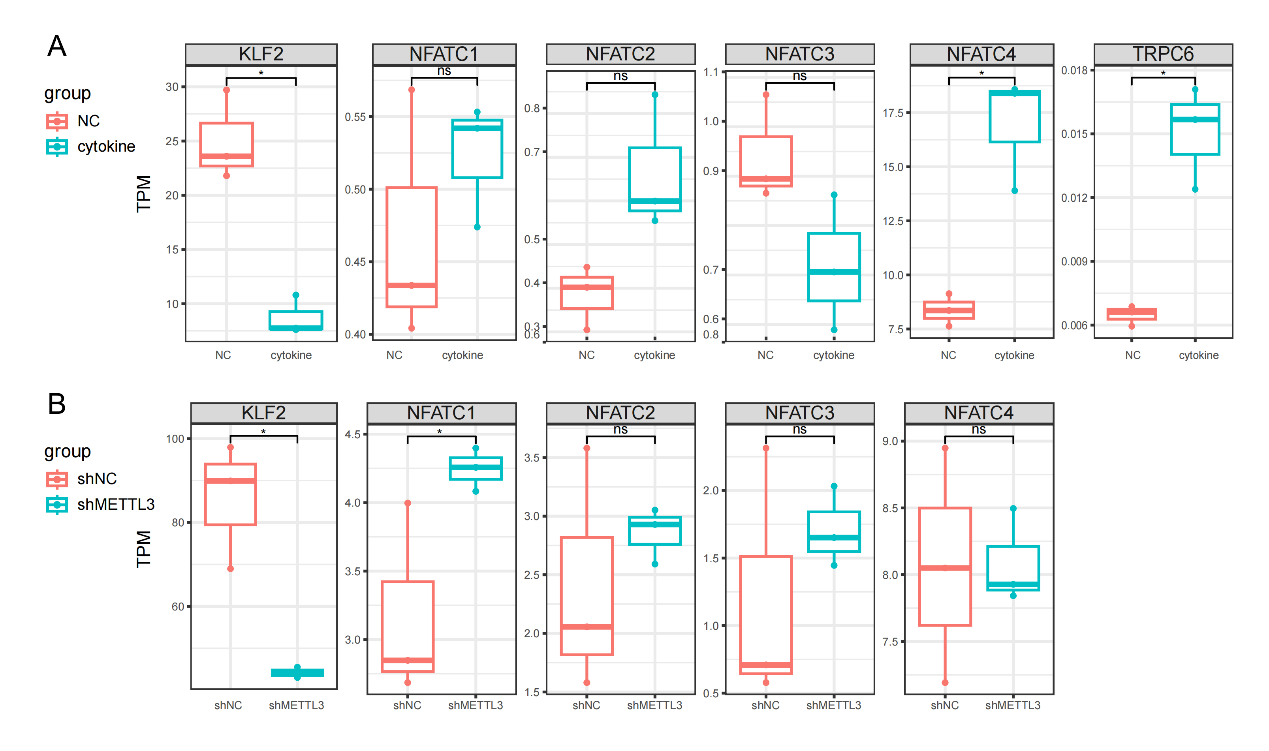
**

**Fig. S4** TRPC6/calcineurin/NFAT pathway in cytokine-treated and METTL3-silenced hPAECs. **A**, **B** The relative expression levels of KLF2, NFATC1, NFATC2, NFATC3, NFATC4, and TRPC6 in transcriptome analysis of cytokine-treated (**A**) and METTL3-silenced (**B**) hPAECs.
